# Comparison of multiple and novel measures of dietary glycemic carbohydrate with insulin resistant status in older women

**DOI:** 10.1186/1743-7075-7-25

**Published:** 2010-04-07

**Authors:** Therese A O'Sullivan, Alexandra P Bremner, Sheila O'Neill, Philippa Lyons-Wall

**Affiliations:** 1 Institute of Health and Biomedical Innovation, Queensland University of Technology, Kelvin Grove, QLD, Australia; 2 School of Population Health, University of Western Australia, Perth, WA, Australia; 3 The Betty Byrne Henderson Research Centre, Royal Brisbane and Women's Hospital, Herston, QLD, Australia; 4 School of Health Sciences, University of Wollongong, Wollongong, NSW, Australia

## Abstract

**Background:**

Previous epidemiological investigations of associations between dietary glycemic intake and insulin resistance have used average daily measures of glycemic index (GI) and glycemic load (GL). We explored multiple and novel measures of dietary glycemic intake to determine which was most predictive of an association with insulin resistance.

**Methods:**

Usual dietary intakes were assessed by diet history interview in women aged 42-81 years participating in the Longitudinal Assessment of Ageing in Women. Daily measures of dietary glycemic intake (n = 329) were carbohydrate, GI, GL, and GL per megacalorie (GL/Mcal), while meal based measures (n = 200) were breakfast, lunch and dinner GL; and a new measure, GL peak score, to represent meal peaks. Insulin resistant status was defined as a homeostasis model assessment (HOMA) value of >3.99; HOMA as a continuous variable was also investigated.

**Results:**

GL, GL/Mcal, carbohydrate (all P < 0.01), GL peak score (P = 0.04) and lunch GL (P = 0.04) were positively and independently associated with insulin resistant status. Daily measures were more predictive than meal-based measures, with minimal difference between GL/Mcal, GL and carbohydrate. No significant associations were observed with HOMA as a continuous variable.

**Conclusion:**

A dietary pattern with high peaks of GL above the individual's average intake was a significant independent predictor of insulin resistance in this population, however the contribution was less than daily GL and carbohydrate variables. Accounting for energy intake slightly increased the predictive ability of GL, which is potentially important when examining disease risk in more diverse populations with wider variations in energy requirements.

## Background

The glycemic index (GI) and glycemic load (GL) are measures of dietary glycemic carbohydrate designed to quantify the rate of digestion and absorption of carbohydrate foods, therefore representing the ability of foods to raise blood glucose concentrations. Derived empirically from feeding studies in humans, the GI is a ranking of the postprandial blood glucose response expressed as a percentage of the response to a reference food (glucose or white bread) containing the same carbohydrate content [1]. Both GI and amount of carbohydrate influence postprandial glucose and insulin excursions, and the GL incorporates both the GI and carbohydrate content of the food to improve the predictive ability of the measure by taking serve size into consideration [2]. Foods higher in GI, carbohydrate or GL result in greater postprandial increases in blood glucose and insulin concentrations [3].

Persistent hyperglycemia from high glycemic carbohydrate diets may contribute to excess insulin secretion and subsequent reduced beta-cell function, potentially leading to insulin resistance and diabetes [4]. However, the long-term metabolic impact of sustained low dietary glycemic intakes in practical prevention of these conditions is controversial [5]. A meta-analysis showed that diets with a high GI or GL independently increased the risk of type 2 diabetes by 40% and 27%, respectively [6], although not all studies were supportive [7-9] and the American Diabetes Association has stated that there is insufficient information to claim that diets lower in dietary glycemic intake reduce diabetes risk [10]. Further, it is not yet clear whether low GI or GL diets have any added advantage over low carbohydrate diets, or whether diets with a high amount of carbohydrate from low GI foods have more metabolic benefits than one of similar GL with a low amount of carbohydrate from high GI foods [11]. In people with type 2 diabetes, a comparison of low carbohydrate versus low GI diets found subjects following the diet lower in carbohydrate showed greater improvements than those on the low GI diet, although both diets resulted in improvements in glycemic control [12].

A possible limitation of previous observational studies has been the use of average daily measures of GI and GL. Implicit in the use of these measures is the assumption that development of insulin resistance is most likely associated with the dietary glycemic potential averaged over the day. However, this measure can conceal variations in glycemic peaks at different meal times. The risk of complications associated with elevated blood glucose and insulin concentrations may be more dependent on the magnitude of postprandial excursions in blood glucose per meal, described as hyperglycemic spikes [13], rather than the average daily glycemic response.

We hypothesised that a measure that accounted for peaks in meal glycemic carbohydrate would be a stronger predictor of insulin resistance than measures averaged over the day. To test this hypothesis, we explored alternative measures of dietary glycemic carbohydrate, including a new measure, the GL peak score, to determine which had the strongest cross-sectional association with insulin resistance in a group of older women. To our knowledge, no prior epidemiological studies have compared the associations between insulin resistance and dietary glycemic carbohydrate on a daily and meal basis.

## Methods

### Study population

Subjects were 511 women aged 42-81 years participating in the Longitudinal Assessment of Ageing in Women (LAW), an age-stratified, multidisciplinary study conducted at Royal Brisbane and Women's Hospital in Brisbane, Australia. Details have been published previously [14]. Data for the current study were collected in year three of LAW. Subjects were excluded from the study if they were confirmed by the study clinician to have diabetes based on self-report, use of medication, and/or fasting glucose concentrations (>6.0 mmol/L) [15]. Subjects were also excluded if less than 85% of their carbohydrate intake could be allocated a GI value, if energy intake was unfeasible (ratio of estimated energy intake to estimated energy expenditure of <0.76) [16,17], or if they did not provide a fasting blood sample. Study procedures were approved by the Human Research Ethics Committees of Queensland University of Technology and Royal Women's Hospital. Subjects gave informed written consent.

### Assessment of dietary intake

Usual dietary intake was assessed by a dietitian during a standardised diet history interview. This method can also assess the pattern of food intake throughout the day, provide an opportunity for the dietitian to clarify issues, and is an appropriate method for investigating associations with retrospective dietary intake and chronic disease status [18,19]. Subjects described the amount and type of foods and drinks consumed in a typical month over the previous six months. Details were obtained on the pattern of food intake throughout the day, with a special focus on carbohydrate-containing items, including brand names and preparation methods. Food models and measuring displays were used to assist in determination of usual serve sizes. Data were analysed using the Foodworks dietary analysis program (Professional Version 4.00, Xyris Software, Brisbane) and the Australian Food and Nutrient Database, combined with a customised GI database comprising published and estimated GI values [20,21]. GI values were able to be allocated to 89% of the total carbohydrate consumed.

Dietary glycemic intake was assessed using nine measures: carbohydrate as a percentage contribution to total energy (carbohydrate percentage), carbohydrate (g/day), dietary GI, GL, GL per megacalorie (GL/Mcal), breakfast GL, lunch GL, dinner GL and GL peak score. Previous analyses of the data showed a significant association between GL and insulin resistance but no significant association with GI [22]. Therefore, the focus of this paper was on alternative measures of GL.

Dietary GI was calculated as the product of the GI and carbohydrate content for each food or beverage, summed for all items consumed and divided by total carbohydrate intake. GL was calculated as the product of the GI and carbohydrate content for each food or beverage, summed for all items [1]. GL/Mcal was calculated by dividing GL by total energy intake in megacalories. To calculate GL peak score, breakfast, morning tea, lunch, afternoon tea and dinner/supper meal GL values were averaged to give a mean meal GL for each subject. GL peaks were identified as GL meal values that were above the mean, and the GL peak score was calculated as the sum of the differences between each GL peak and the mean meal GL. For example, if average meal GL values are 5, 0, 9, 1 and 10 for breakfast, morning tea, lunch, afternoon tea and dinner, respectively, dietary GL is the sum of the five meal GL values or 25, and therefore the mean meal value is 5. GL peaks over the mean occur at lunch and dinner, and exceed the mean by 4 and 5, respectively. Therefore the GL peak score, which represents the sum of the peak values, is 9.

### Assessment of insulin resistance

Insulin resistance was measured using the homeostasis model assessment (HOMA), where HOMA = fasting plasma insulin (mU/L) × fasting plasma glucose (mmol/L)/22.5, as validated against the glucose clamp technique [23]. Insulin resistant status was defined using a cut-off point of HOMA ≥ 3.99 [24], which has been previously evaluated in this population [22].

### Confounding variables

Menopausal and hormone therapy (HT) status was grouped as: premenopausal; using HT for ≥ 12 months; or peri- or postmenopausal and using HT for <12 months. Family history was defined as a first-degree blood relative diagnosed with type 1 or 2 diabetes mellitus (yes/no). Smoking was assessed in pack years and as smoking status (never, current, ex-smoker). Physical activity was classified as one of six levels based on incidental and purposeful exercise [25] and reclassified into two levels (active: walk or other activity ≥ 2/week; sedentary: walk or other activity <2/week), after statistical analyses showed no significant differences between using two or six levels. Anthropometric measures were collected by a trained operator using standard methodology [26]; waist and hip circumferences were measured to the nearest 0.1 cm, height was assessed to the nearest 0.1 cm with a stadiometer (Holtain Ltd, Crymych, UK), and weight was measured to the nearest 0.01 kg using a Seca standing scale (BPS Instruments, US). Body mass index (BMI) was calculated as weight (kg) divided by height squared (m^2^). Average daily intakes of saturated fat, alcohol, dietary fibre and energy were obtained from the diet history.

### Statistical analysis

Subject characteristics were compared using independent t-tests and chi-square tests. Nutrient intakes are reported as mean ± standard deviation. Independent t-tests were used to evaluate differences between dietary glycemic intakes based on insulin resistant status. Bivariate correlations were used to investigate relationships between the different measures of dietary glycemic intake. Logistic regression models included the following potential confounding variables, based on examination of our data and previous research: age, age squared, BMI, waist circumference, family history of diabetes, HT status, physical activity level, and intakes of energy, alcohol, dietary fibre and saturated fat. Odds ratios were used to describe the association with the glycemic variables on a per-unit basis. Results of odds ratios cannot be directly compared across glycemic variables due to different units of measure. Therefore, Nagelkerke's R^2 ^coefficients were used to compare the contributions from the different glycemic measures. The R^2 ^value was noted in the full model that contained potential confounding variables as well as the dietary glycemic intake variable of interest, and was compared to the baseline R^2 ^value of 0.529 for the model with the glycemic variable removed. R^2 ^values represent the ability of the model to explain the variability of the outcome, and range from 0 to 1, with larger values indicating a better fit [27]. Standardised beta coefficients for each glycemic variable were calculated by multiplying the beta coefficient obtained from the logistic regression model by the standard deviation of the variable, allowing comparison per standard deviation. Multivariate linear regression models were used to investigate associations between glycemic variables and the continuous measure of HOMA. Log HOMA was used due to the skewed distribution of HOMA values.

A receiver operating characteristic curve [28] was used to estimate sensitivity and specificity of the model with the highest predictive ability. To produce a parsimonious model for prediction, the maximal logistic regression model for the most predictive dietary glycemic intake variable was reduced by removing the least significant variable and refitting the model until all remaining variables were significant at the 5% level. When insignificant variables were removed from the model, the change in R^2 ^coefficient was checked to ensure removal of the variable had a minimal effect on the overall model, and variables that had been removed were retested in the parsimonious model.

Statistical analyses were conducted using the Statistical Package for Social Sciences (SPSS) for Windows (release 14.0) with statistical significance set to 5%.

## Results

### Subjects

Of the 511 subjects who commenced the LAW study, 470 completed a diet history for the current study. Reasons for non-completion were: unable to attend appointment (n = 24), illness (n = 13), declined to participate (n = 2), and death (n = 2). Of the subjects who completed the diet history, 329 subjects had complete clinical, sociodemographic and lifestyle data and were included in the analysis of GI, GL, GL/Mcal, carbohydrate and carbohydrate percentage. Reasons for exclusion were: identified as an under-reporter (n = 11), did not meet the criterion of ≥ 85% of carbohydrate able to be allocated a GI value (n = 90), had diabetes (n = 23), and did not give a fasting blood sample (n = 17). Characteristics of subjects in the included (n = 329) and excluded (n = 182) groups did not differ significantly except for BMI and waist to hip ratio, which were larger in the excluded group (Table 1). Of the 329 subjects, 200 had GL data for each meal (≥ 85% of carbohydrate consumed per meal allocated GI values) and were used to analyse breakfast, lunch and dinner GL, and GL peak score. Subjects in the n = 329 and n = 200 groups were similar in age (61.6 ± 10.4 versus 61.8 ± 10.3 years), BMI (26.1 ± 4.4 versus 26.6 ± 4.9 kg/m^2^), prevalence of insulin resistance (11.0% versus 10.5%) and energy intake (2.01 ± 0.31 versus 2.02 ± 0.31 Mcal), respectively. Within the n = 329 group, there were no significant differences in BMI, waist to hip ratio, activity level, HT status, smoking status between subjects included in the n = 200 compared with the 129 excluded (data not shown).

**Table 1:** Characteristics of subjects in the LAW study (n = 511)

Characteristic	Included in glycemic intake analysis (n = 329)	Not included in glycemic intake analysis (n = 182)	P
Age (years) (mean ± SD)	61.7 ± 10.4	60.7 ± 11.0	0.37
Activity level (valid %)^†^			
Active (walk or other activity ≥ 2/week)	66.0	63.9	0.68
Sedentary (walk or other activity <2/week)	34.0	36.1	
Missing (n)	2	27	
Menopausal and hormone therapy (HT) status (valid %)^†^			
Premenopausal	12.8	12.2	0.95
Using HT ≥ 12 months	43.8	46.3	
Peri or postmenopausal, and using HT <12 months	43.4	41.5	
Missing (n)	9	141	
Smoking status (valid %)^†^			
Non-smoker	54.7	53.7	0.78
Ex-smoker	36.4	34.1	
Current smoker	8.9	12.2	
Missing (n)	2	141	
Anthropometry (mean ± SD)			
Body mass index (kg/m^2^)	26.5 ± 4.8	28.2 ± 5.8	<0.01
Waist to hip ratio	0.80 ± 0.1	0.81 ± 0.1	0.03

^†^Valid % expresses the proportion of subjects with this characteristic when missing values are removed from both groups

### Dietary glycemic carbohydrate

Scatter plots of glycemic carbohydrate variables against raw HOMA scores (unadjusted for confounding variables) can be viewed in Additional File 1. Mean intakes for all dietary glycemic carbohydrate variables were higher in subjects with, compared to without, insulin resistance; differences were significant for GL/Mcal, GL, carbohydrate percentage, carbohydrate (g) and lunch GL, and remained significant after adjusting for potential confounding variables in separate logistic regression models (Table 2). There were no significant differences in GL peak score between groups, however this variable was significantly associated with insulin resistance after adjusting for confounding factors. Correlations between dietary glycemic variables were highest for dietary GL versus carbohydrate (r = 0.92), GL/Mcal versus carbohydrate percentage (r = 0.86), GL/Mcal versus GL (r = 0.72), and GL peak score versus dinner GL (r = 0.72) (all P < 0.01). The correlation between GL peak score and GL was lower (r = 0.42, P < 0.01).

**Table 2:** Comparison of nine dietary glycemic intake variables and their relative contribution to prediction of insulin resistant status in LAW study subjects (n = 200)

Glycemic variable	Mean glycemic intake^†^		Odds of insulin resistance^§^	Nagelkerke's R^2 ^coefficient^¶^		
	**Subjects with insulin resistance (n = 19)**	**Subjects with-out insulin resistance (n = 181)**	**Odds ratio (95%CI)**	**R^2 ^value with variable**	**Increase in R^2 ^value**	***P *value**

None (baseline)				0.529	n/a	n/a
Daily glycemic measures:						
GL per megacalorie	68.1 ± 9.1	58.2 ± 9.1**	1.21 (1.09-1.36)**	0.674	0.145	<0.01
GL	141 ± 33	117 ± 23**	1.10 (1.04-1.16)**	0.671	0.142	<0.01
Carbohydrate percentage	52.6 ± 5.1	46.6 ± 6.5**	1.36 (1.13-1.63)**	0.670	0.141	<0.01
Carbohydrate (g)	265 ± 54	227 ± 43**	1.06 (1.03-1.10)**	0.668	0.139	<0.01
GI	56.8 ± 3.4	55.9 ± 4.8	1.09 (0.91-1.29)	0.536	0.007	0.35
Meal glycemic measures:						
GL peak score	38.9 ± 20.0	32.4 ± 10.8	1.06 (1.00-1.12)*	0.568	0.039	0.04
Lunch GL	33.4 ± 13.3	27.2 ± 9.5*	1.07 (1.00-1.14)*	0.566	0.037	0.04
Dinner GL	48.5 ± 25.1	41.8 ± 12.8	1.05 (0.99-1.10)	0.556	0.027	0.09
Breakfast GL	36.0 ± 12.7	30.5 ± 11.7	1.05 (0.99-1.13)	0.549	0.020	0.13

^†^Independent t-tests used to compare subjects with and subjects without insulin resistance (defined as HOMA ≥ 3.99).

*P < 0.05, **P < 0.01.

^§ ^Logistic regression adjusted for age, age squared, waist circumference, body mass index, physical activity level, family history of diabetes, menopausal and hormone therapy status, saturated fat, alcohol, dietary fibre and energy.

^¶ ^Relative contributions to the predictive model of insulin resistant status, as measured by the increase in Nagelkerke's R^2 ^coefficient when individual glycemic variables were included in the logistic regression model with potential confounding factors. A larger increase in R^2 ^compared to baseline value indicates greater prediction of insulin resistance in the model.

HOMA: homeostasis model assessment for insulin resistance. Glycemic variables are defined in the Methods section. GL: glycemic load, GI: glycemic index.

Of the nine measures, GL/Mcal contributed the most to prediction of insulin resistant status with the largest increase in Nagelkerke's R^2^coefficient. Significant associations were also seen with GL, carbohydrate percentage, carbohydrate (g), GL peak score and lunch GL (Table 2). Comparison of standardised beta coefficients showed that carbohydrate (g), GL, carbohydrate percentage and GL/Mcal had the greatest impact per increase of one standard deviation, with standardised beta coefficients of 1.8 and above; peak meal GL, lunch GL, dinner GL and breakfast GL had standardised beta coefficients of 0.7 and below (data not shown). No glycemic variables were significantly associated with HOMA as a continuous variable in multivariate linear regression models (Table 3). Comparison of the standardised beta coefficients suggests that the four highest glycemic variable contributors to the linear regression models were the same as those in the logistic regression models, but in the reverse order; carbohydrate (g) was the highest contributor per standard deviation, although differences between variables were small.

**Table 3:** Comparison of nine glycemic variables in multivariate linear regression models (n = 200) for prediction of log HOMA^† ^

	Unstandardized Coefficients		Standardized Coefficients	
		
Glycemic variable	B	Std. Error	Beta	P
Daily glycemic measures:				
GL per megacalorie	0.006	0.005	0.084	0.20
GL	0.003	0.002	0.100	0.22
Carbohydrate percentage	0.014	0.008	0.132	0.06
Carbohydrate (g)	0.002	0.002	0.146	0.13
GI	-0.007	0.010	-0.047	0.47
Meal glycemic measures:				
GL peak score	-0.001	0.004	-0.021	0.72
Lunch GL	0.003	0.004	0.048	0.44
Dinner GL	0.000	0.003	-0.007	0.91
Breakfast GL	0.002	0.004	0.040	0.54

^†^Logistic regression adjusted for age, age squared, waist circumference, body mass index, physical activity level, family history of diabetes, menopausal and hormone therapy status, saturated fat, alcohol, dietary fibre and energy.

HOMA: homeostasis model assessment for insulin resistance. Glycemic variables are defined in the Methods section. GL: glycemic load, GI: glycemic index.

### Models of insulin resistant status using GL per megacalorie

GL/Mcal was selected to develop a model of insulin resistant status. Significant variables retained in the parsimonious model (n = 329) were age, age squared, waist circumference, physical activity, energy intake and GL/Mcal. The equation generated for the logistic regression model was: Log (P/1-P) = -58.48 + 1.032 × age - 0.007432 × age^2 ^+ 0.1068 × waist circumference - 1.274 × physical activity level + 2.292 × daily energy intake + 0.1186 × GL/Mcal, where P equals the probability of being insulin resistant. A receiver operating characteristic curve was generated to measure how accurately the model categorised subjects into insulin resistant or non-insulin resistant status with optimal sensitivity and specificity [28]. The P value in the odds equation that had the smallest difference between sensitivity and specificity with the highest possible result was 0.0983, assuming equal importance of sensitivity and specificity. This point corresponded to a sensitivity of 0.83 and specificity of 0.86.

**Figure 1:**
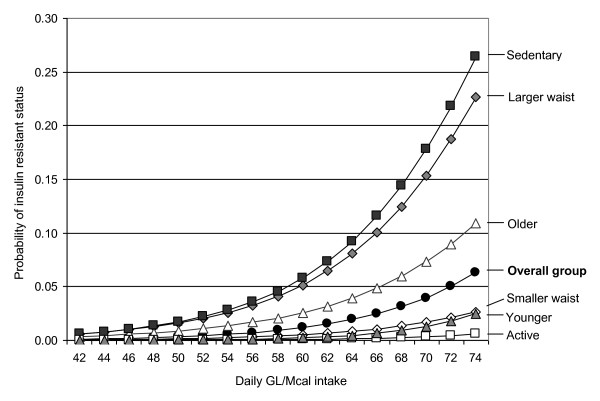
**Predictive model of insulin resistance in LAW study women (n = 329) at differing intakes of GL per megacalorie (GL/Mcal)**. The predictive model uses average values in subgroups of women who were younger (≤ 61 years) or older (>61 years), sedentary (exercise frequency <2/week) or active (exercise frequency ≥ 2/week), and with smaller waist circumference (≤ 88 cm) or larger waist circumference (>88 cm), in comparison with the overall group (n = 329). Variables for the models are based on mean subject values, with an age of 61.7 years, waist circumference of 83.7 cm, physical activity level classification of active, and energy intake of 2.01 Mcal.

To investigate the model further, mean values for covariates were placed in the logistic regression equation and variation in GL/Mcal was set from 42 to 74 to represent the middle 90% of values in the population. For the subject group (n = 329) with mean age 61.7 years, waist circumference 83.7 cm, energy intake 2.01 Mcal, and the most frequent physical activity classification (active), the curve predicted that probability of an association with insulin resistant status increased from 0.1% at an intake of 42 GL/Mcal to 5.5% at an intake of 74 GL/Mcal (Figure 1). The probability equation was also used to investigate heterogeneity of GL/Mcal for different subgroups of the study population. Curves generated within the younger (≤ 61 years)/older (>61 years), sedentary (exercise frequency <2/week)/active (exercise frequency ≥ 2/week) and smaller waisted (≤ 88 cm)/larger waisted (>88 cm) showed similar shapes to the curve for the overall group. The steepest gradient of risk was observed in sedentary subjects, with the probability of insulin resistance increasing from 0.6% at intakes of 42 GL/Mcal to 26.5% at intakes of 74 GL/Mcal. In active subjects the probability of insulin resistance increased from <0.1% to 0.6% over the same range of intakes and in subjects with larger waist circumferences the probability increased from 0.6% to 22.7% (Figure 1).

## Discussion

We explored multiple and novel measures of dietary glycemic carbohydrate in a group of older Australian women to provide insight into which variables were most predictive of an association with insulin resistance. Mean intakes for all glycemic measures were consistently higher in women who were insulin resistant compared to those who were not insulin resistant. Contrary to our hypothesis however, the largest contributors to prediction of insulin resistance, independently of other risk factors, were the daily glycemic measures rather than meal based measures (Table 2). Our data suggest GL/Mcal was the glycemic variable that had the highest contribution to the prediction of insulin resistance. Adjusting for overall energy intake, in the form of GL/Mcal, increased the predictive ability with insulin resistant status compared to the GL unadjusted. This could be especially important when examining dietary glycemic intake in relation to disease risk in more diverse population groups with wider variations in energy requirements due to gender, age or physical activity. The GL/Mcal became more clinically important in subjects who were older, sedentary or had a larger waist circumference, as these factors potentiated the effect (Figure 1).

The difference in predictive ability of the four highest contributing glycemic variables (GL/Mcal, GL, carbohydrate (g) and carbohydrate percentage) was minor, with a separation of only 0.006 in Nagelkerke's R^2 ^coefficients between the first and fourth placed variables. These four glycemic variables were also the highest contributors per standard deviation to prediction of HOMA as a continuous variable. As carbohydrate is used to calculate GL, this was partly due to colinearity between the measures. The advantage of using the GL over carbohydrate in prediction of insulin resistance in this population is slight. Therefore in practice, the prescription of carbohydrate reduction may be simpler to follow than reducing GL, as long as intakes of dietary fibre, vitamins, and minerals remain nutritionally adequate. The type of fat used to replace carbohydrate should also be considered, as an increase in saturated fat has the potential to raise low-density lipoprotein cholesterol [29].

The lack of significant associations when using HOMA as a continuous variable suggests that relationships between insulin resistance and glycemic intake variables may be more noticeable when subjects with higher HOMA values are identified as a separate group. Causality cannot be inferred due to the cross-sectional design, however our results support further investigation of the effect of dietary glycemic intake on development of insulin resistance.

A unique aspect of this study was investigation of the association between insulin resistance and dietary glycemic carbohydrate on a meal basis. Post-prandial glucose response can vary with the time of day. In previous studies, plasma glucose concentrations were higher after breakfast than lunch, despite a lower GL for the breakfast meal [30] or when both breakfast and lunch contained the same type and amount of cereal [31]. In our study, lunch GL but not breakfast or dinner GL, was a significant predictor of insulin resistance when adjusted for confounding factors. The relevance of lunch per se is not clear; however evidence for the possible importance of lunch in relation to risk of chronic disease was also seen in a five-year prospective study, in which blood glucose concentrations after lunch predicted cardiovascular events in women with diabetes [32].

We also used the analysis of GL on a meal basis to conceptualise the GL peak score, a new measure designed to represent the magnitude of glycemic peaks at meals during the day. The magnitude of postprandial hyperglycemia has been shown to influence the degree of oxidation of low-density lipoprotein cholesterol in subjects with diabetes [33] and oxidative stress in healthy subjects [34]. For comparison with a disease outcome such as insulin resistance, it was hypothesised that a measure of hyperglycemic spikes could be a more relevant predictor of the glycemic potential than the daily measures used in previous studies. We designed the GL peak score to express the magnitude of GL peaks during the day, relative to each subject's mean dietary GL, and to quantify high peaks across all meals, not just the single meal with the highest peak. While our data indicated that a dietary pattern consisting of relatively high peaks was a significant independent predictor of insulin resistance, the contribution was less than that of the dietary GL. Depending on the degree of fluctuation of meal GL values, a subject with a high GL peak score can have a low or a high dietary GL, as evident in the relatively low correlation we observed between these two measures (r = 0.42). However, the potential impact of these alternatives on insulin resistance is not clear from our data. The use of mean meal GL to calculate the GL peak score may also have misrepresented subjects who alternated between missing meals and consuming large meals (for example, at breakfast). While the technique we developed to calculate GL peak score was carefully considered, further research may suggest another technique that provides a more optimal measure of hyperglycemic peaks for comparison with chronic disease status.

A strength of our study was the comprehensive assessment of usual dietary glycemic intake by diet history interviews. This allowed us to quantify individual carbohydrate serve sizes to optimise the precision in our estimates of dietary glycemic carbohydrate, and provided data on meal patterns. A possible limitation to application of this research to the wider population is that the BMI and waist circumference of subjects in this analysis were lower than other subjects in the larger LAW study (Table 1), in part due to exclusion of women with diabetes for the current analyses.

## Conclusion

Of the six glycemic variables that were significant predictors of insulin resistant status, there was little difference between dietary GL, either corrected or uncorrected for energy, dietary carbohydrate or carbohydrate expressed as a percentage of total energy. Due to the greater association with these daily measures compared with meal measures such as GL peak score and lunch GL, we conclude that insulin resistance in the LAW women was more closely associated with average dietary glycemic variables than with glycemic variables representing individual meal variations. Although GL/Mcal was the highest contributor to prediction of insulin resistant status in logistic regression models, the daily carbohydrate variables showed similar values, and performed slightly better in prediction of insulin resistance in linear regression models using HOMA as a continuous variable. Our results suggest that in this population of older women, the potential benefit to insulin resistance offered by a lower GL diet may be similar to lowering daily carbohydrate intake, assuming nutritional targets are met for other dietary aspects.

## Abbreviations Used

GI: Glycemic index; GL: glycemic load; GL/Mcal: GL per megacalorie; LAW: Longitudinal Assessment of Ageing in Women; HOMA: homeostasis model assessment.

## Competing interests

The authors declare that they have no competing interests.

## Authors' contributions

TO'S: study conception and design, acquisition of dietary data, analysis and interpretation of data, manuscript drafts; AB: analysis and interpretation of data, manuscript revision; SO'N: conception and design of LAW study, acquisition of clinical data, clinical director of LAW study, manuscript revision; PLW: study conception and design, interpretation of data, manuscript revision. All authors read and approved the final manuscript.

## Supplementary Material

Additional file 1 Scatter plots of glycemic carbohydrate variables against HOMA Click here for file

## References

[B1] Jenkins DJ Wolever TM Taylor RH Barker H Fielden H Baldwin JM Bowling AC Newman HC Jenkins AL Goff DV Glycemic index of foods: a physiological basis for carbohydrate exchange Am J Clin Nutr 1981 34 362 366 6259925 10.1093/ajcn/34.3.362

[B2] Salmeron J Ascherio A Rimm E Colditz G Spiegelman D Jenkins D Stampfer M Wing A Willett W Dietary fiber, glycemic load, and risk of NIDDM in men Diabetes Care 1997 20 545 550 10.2337/diacare.20.4.545 9096978

[B3] Holt SH Miller JCB Petocz P An insulin index of foods: The insulin demand generated by 1000-kj portions of common foods Am J Clin Nutr 1997 66 1264 1276 9356547 10.1093/ajcn/66.5.1264

[B4] Wolever TMS Dietary carbohydrates and insulin action in humans Br J Nutr 2000 83 S97 S102 10.1017/S0007114500001021 10889799

[B5] Miles JM A role for the glycemic index in preventing or treating diabetes? Am J Clin Nutr 2008 87 1 2 18175728 10.1093/ajcn/87.1.1

[B6] Barclay AW Petocz P McMillan-Price J Flood VM Prvan T Mitchell P Brand-Miller JC Glycemic index, glycemic load, and chronic disease risk- a meta-analysis of observational studies Am J Clin Nutr 2008 87 627 637 18326601 10.1093/ajcn/87.3.627

[B7] Sahyoun NR Anderson AL Tylavsky FA Lee JS Sellmeyer DE Harris TB Dietary glycemic index and glycemic load and the risk of type 2 diabetes in older adults Am J Clin Nutr 2008 87 126 131 18175745 10.1093/ajcn/87.1.126 PMC2265787

[B8] Lau C Færch K Glümer C Tetens I Pedersen O Carstensen B Jørgensen T Borch-Johnsen K Dietary glycemic index, glycemic load, fiber, simple sugars, and insulin resistance: The Inter99 study Diabetes Care 2005 28 1397 1403 10.2337/diacare.28.6.1397 15920058

[B9] Liese A Schulz M Fang F Wolever T D'Agostino RJ Sparks K Mayer-Davis E Dietary glycemic index and glycemic load, carbohydrate and fiber intake, and measures of insulin sensitivity, secretion, and adiposity in the Insulin Resistance Atherosclerosis Study Diabetes Care 2005 28 2832 2838 10.2337/diacare.28.12.2832 16306541

[B10] American Diabetes Association Nutrition Recommendations and Interventions for Diabetes: A position statement of the American Diabetes Association Diabetes Care 2008 31 S61 78 10.2337/dc08-S061 18165339

[B11] Barclay AW Brand-Miller JC Wolever TMS Glycemic index, glycemic load, and glycemic response are not the same Diabetes Care 2005 28 1839 1840 10.2337/diacare.28.7.1839 15983358

[B12] Westman E Yancy W Mavropoulos J Marquart M McDuffie J The effect of a low-carbohydrate, ketogenic diet versus a low-glycemic index diet on glycemic control in type 2 diabetes mellitus Nutr Metab 2008 5 36 10.1186/1743-7075-5-36 PMC2633336 19099589

[B13] Le Devehat C The concept of "hyperglycemic spike" Diabetes Metab 1997 23 358 359 9432278

[B14] Khoo SK O'Neill S Travers C Oldenburg B the LAW Study Group Age-related changes relevant to health in women: design, recruitment, and retention strategies for the Longitudinal Assessment of Women (LAW) study J Womens Health 2008 17 135 146 10.1089/jwh.2006.0291 18240990

[B15] World Health Organisation Definition, diagnosis and classification of diabetes mellitus and its complications: report of a WHO consultation 1999 Geneva, World Health Organisation

[B16] Black AE The sensitivity and specificity of the Goldberg cut-off for EI: BMR for identifying diet reports of poor validity Euro J Clin Nutr 2000 54 395 404 10.1038/sj.ejcn.1600971 10822286

[B17] Goldberg G Black A Jebb S Cole T Murgatroyd P Coward W Prentic A Critical evaluation of energy intake data using fundamental principles of energy physiology: Derivation of cut-off limits to identify under-reporting Euro J Clin Nutr 1991 45 569 581 1810719

[B18] Tapsell LC Brenninger V Barnard J Applying conversation analysis to foster accurate reporting in the diet history interview J Am Diet Assoc 2000 100 818 824 10.1016/S0002-8223(00)00237-6 10916521

[B19] Jain M Diet history: Questionnaire and interview techniques used in some retrospective studies of cancer J Am Diet Assoc 1989 89 1647 1652 2809042

[B20] University of Sydney Home of the Glycemic Index - GI Database http://www.glycemicindex.com/accessed 2005-2007

[B21] Foster-Powell K Holt S Brand-Miller J International table of glycemic index and glycemic load values: 2002 Am J Clin Nutr 2002 76 5 56 12081815 10.1093/ajcn/76.1.5

[B22] O'Sullivan TA Bremner AP O'Neill S Lyons-Wall P Glycemic load is associated with insulin resistance in older Australian women Eur J Clin Nutr 2010 64 80 87 10.1038/ejcn.2009.115 19756025

[B23] Bonora E Targher G Alberiche M Bonadonna R Saggiani F Zenere M Monauni T Muggeo M Homeostasis model assessment closely mirrors the glucose clamp technique in the assessment of insulin sensitivity: studies in subjects with various degrees of glucose tolerance and insulin sensitivity Diabetes Care 2000 23 57 63 10.2337/diacare.23.1.57 10857969

[B24] Wahrenberg H Hertel K Leijonhufvud B-M Persson L-G Toft E Arner P Use of waist circumference to predict insulin resistance: retrospective study Br Med J 2005 330 1363 1364 10.1136/bmj.38429.473310.AE PMC558285 15833749

[B25] Hirvensalo M Lintunen T Rantanen T The continuity of physical activity - a retrospective and prospective study among older people Scand J Med Sci Sports 2000 10 37 41 10.1034/j.1600-0838.2000.010001037.x 10693611

[B26] Gibson R Nutritional Assessment: A Laboratory Manual 1993 New York, Oxford University Press

[B27] Nagelkerke NJD A note on a general definition of the coefficient of determination Biometrika 1991 78 691 692 10.1093/biomet/78.3.691

[B28] Altman D Practical Statistics for Medical Research 1991 London, Chapman and Hill

[B29] Hu FB Manson JE Willett WC Types of dietary fat and risk of coronary heart disease: A critical review J Am Coll Nutr 2001 20 5 19 11293467 10.1080/07315724.2001.10719008

[B30] Monnier L Colette C Rabasa-Lhoret R Lapinski H Caubel C Avignon A Boniface H Morning hyperglycemic excursions Diabetes Care 2002 25 737 741 10.2337/diacare.25.4.737 11919134

[B31] Wolever T Bolognesi C Time of day influences relative glycaemic effect of foods Nutr Res 1996 16 381 384 10.1016/0271-5317(96)00019-X

[B32] Cavalot F Petrelli A Traversa M Bonomo K Fiora E Conti M Anfossi G Costa G Trovati M Postprandial blood glucose is a stronger predictor of cardiovascular events than fasting blood glucose in type 2 diabetes mellitus, particularly in women J Clin Endocrinol Metab 2006 91 813 819 10.1210/jc.2005-1005 16352690

[B33] Diwadkar VA Anderson JW Bridges SR Gowri MS Oelgten PR Postprandial low-density lipoproteins in type 2 diabetes are oxidized more extensively than fasting diabetes and control samples Proc Soc Exp Biol Med 1999 222 178 184 10.1046/j.1525-1373.1999.d01-129.x 10564543

[B34] Marfella R Quagliaro L Nappo F Ceriello A Giugliano D Acute hyperglycemia induces an oxidative stress in healthy subjects J Clin Invest 2001 108 635 636 11518739 10.1172/JCI13727 PMC209408

