# Supplementary material for: Comparison of multiple and novel measures of dietary glycemic carbohydrate with insulin resistant status in older women
Source: Nutr Metab (Lond). 2010 Apr 7;7:25. doi: 10.1186/1743-7075-7-25 (PMC2859357; doi:10.1186/1743-7075-7-25)
Supplement: Additional file 1 — Scatter plots of glycemic carbohydrate variables against HOMA [file 1743-7075-7-25-S1.DOC]

**Additional file 1:** Scatter plots of glycemic carbohydrate variables against HOMA^†^


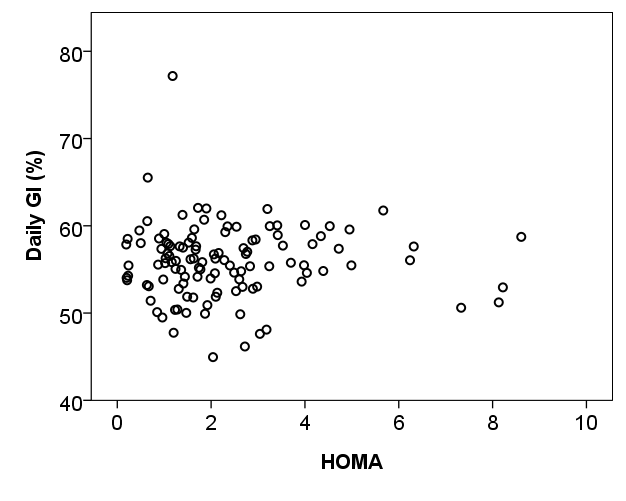


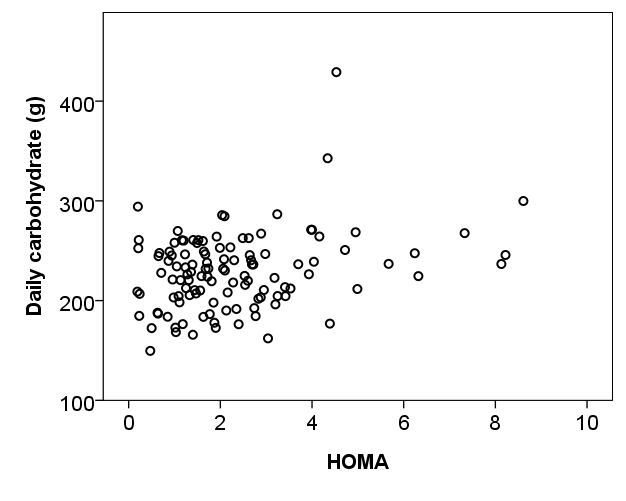


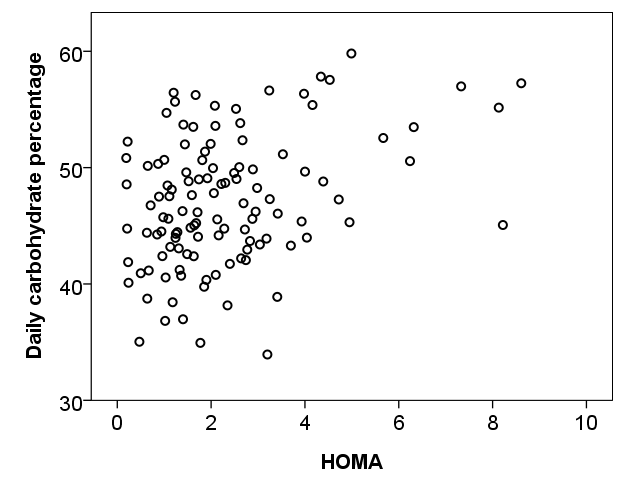


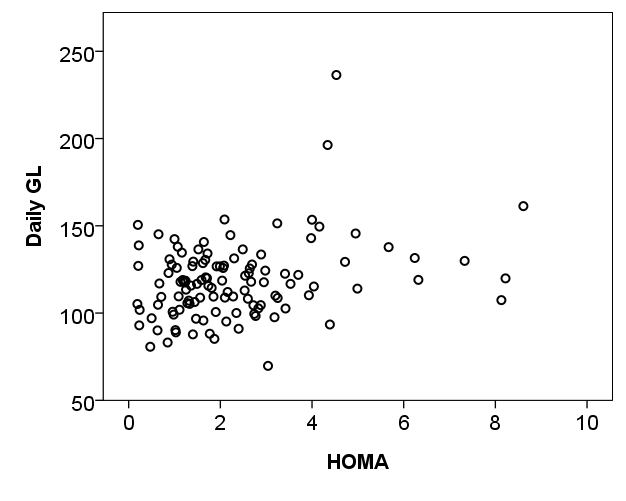


r = -0.02

r =0.09


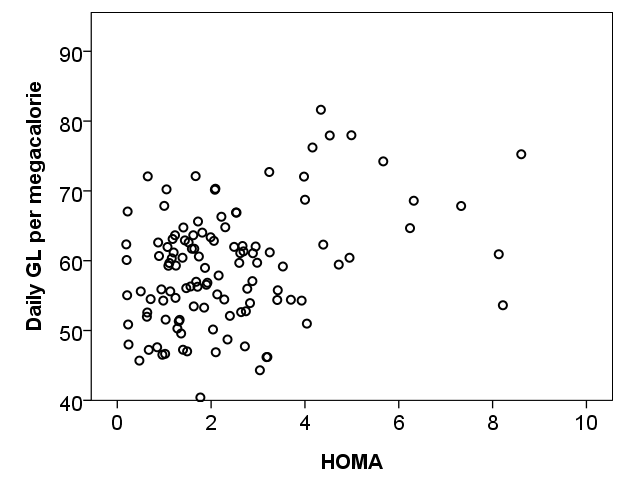


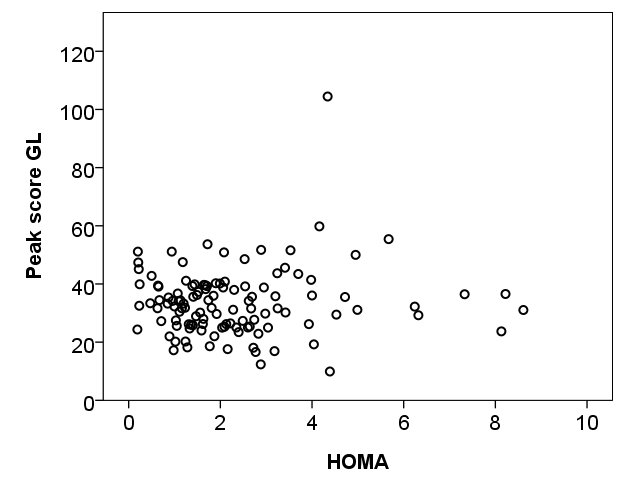


r = 0.07

r = 0.13

r = -0.02

r = 0.12


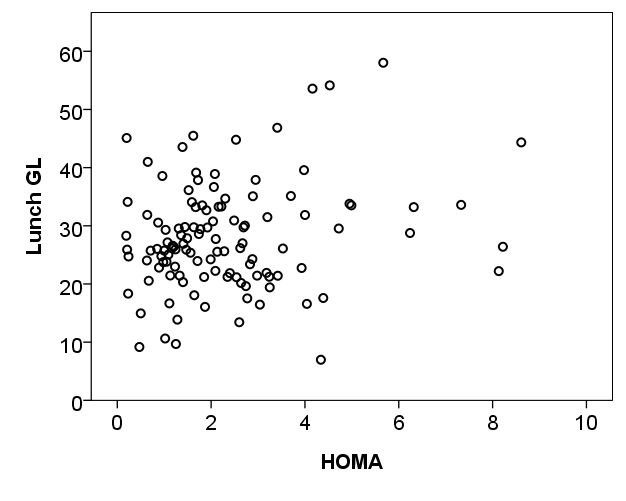


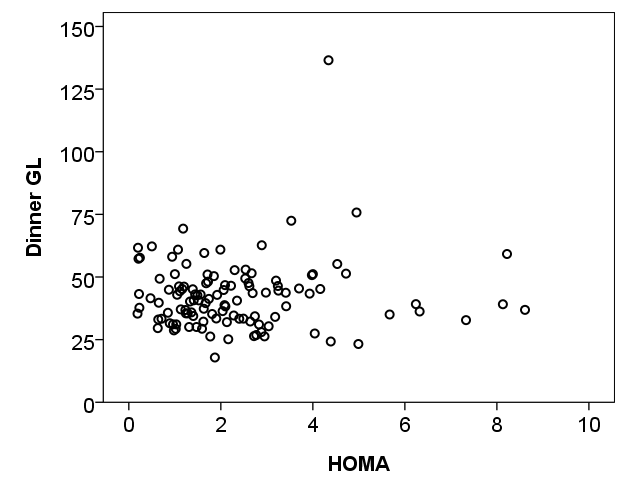


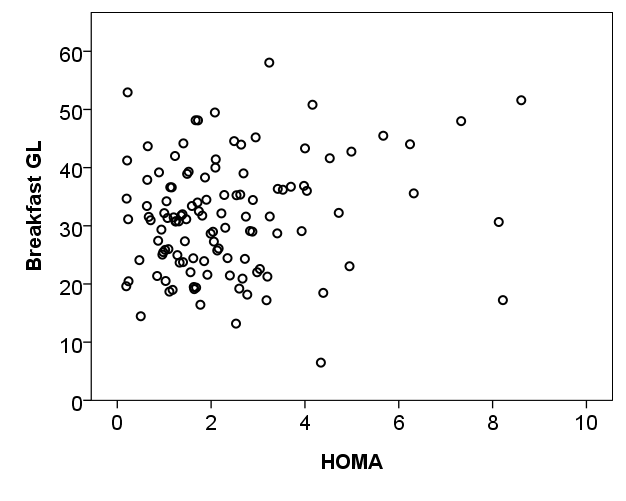


r = 0.09

r = 0.04

r = 0.01

^†^ Correlations using Spearman r to allow for the non-normality of the data. These correlations are unadjusted and do not account for confounding factors. No correlations were significant (P<0.05).

GI = glycemic index, GL = glycemic load, HOMA = homeostasis model assessment
